# Expression and *In Vivo* Rescue of Human ABCC6 Disease-Causing Mutants in Mouse Liver

**DOI:** 10.1371/journal.pone.0024738

**Published:** 2011-09-14

**Authors:** Olivier Le Saux, Krisztina Fülöp, Yukiko Yamaguchi, Attila Iliás, Zalán Szabó, Christopher N. Brampton, Viola Pomozi, Krisztina Huszár, Tamás Arányi, András Váradi

**Affiliations:** 1 Department of Cell and Molecular Biology, John A. Burns School of Medicine, University of Hawaii, Honolulu, Hawaii, United States of America; 2 Institute of Enzymology, Hungarian Academy of Sciences, Budapest, Hungary; University of South Florida College of Medicine, United States of America

## Abstract

Loss-of-function mutations in *ABCC6* can cause chronic or acute forms of dystrophic mineralization described in disease models such as pseudoxanthoma elasticum (OMIM 26480) in human and dystrophic cardiac calcification in mice. The ABCC6 protein is a large membrane-embedded organic anion transporter primarily found in the plasma membrane of hepatocytes. We have established a complex experimental strategy to determine the structural and functional consequences of disease-causing mutations in the human ABCC6. The major aim of our study was to identify mutants with preserved transport activity but failure in intracellular targeting. Five missense mutations were investigated: R1138Q, V1298F, R1314W, G1321S and R1339C. Using *in vitro* assays, we have identified two variants; R1138Q and R1314W that retained significant transport activity. All mutants were transiently expressed *in vivo*, in mouse liver via hydrodynamic tail vein injections. The inactive V1298F was the only mutant that showed normal cellular localization in liver hepatocytes while the other mutants showed mostly intracellular accumulation indicating abnormal trafficking. As both R1138Q and R1314W displayed endoplasmic reticulum localization, we tested whether 4-phenylbutyrate (4-PBA), a drug approved for clinical use, could restore their intracellular trafficking to the plasma membrane in MDCKII and mouse liver. The cellular localization of R1314W was significantly improved by 4-PBA treatment, thus potentially rescuing its physiological function. Our work demonstrates the feasibility of the *in vivo* rescue of cellular maturation of some ABCC6 mutants in physiological conditions very similar to the biology of the fully differentiated human liver and could have future human therapeutic application.

## Introduction

Commonly found in aging tissue, dystrophic calcification is defined as the abnormal deposition of calcium salts in altered or diseased tissues. It also occurs in pathologies such as diabetes, hypercholesterolemia, chronic renal failure and certain genetic conditions. Under pathological conditions, this abnormal mineralization can occur in response to metabolic, mechanical, infectious, or inflammatory injuries and its etiology is heterogeneous with overlapping yet distinct molecular mechanisms of initiation and progression. Pseudoxanthoma elasticum (PXE, OMIM 26480) in human and dystrophic cardiac calcification (DCC) in mice are similar pathologies both defined by dystrophic mineralization of cardiovascular, ocular and dermal tissues. Both conditions derive from loss-of-function mutations in the human *ABCC6* and mouse *Abcc6* genes [1], [2], [3], [4], [5]. PXE is characterized by dystrophic calcification primarily affecting elastic fibers in skin, arteries and the Bruch's membrane of the eye [6], [7], [8]. The causality of mutations of the *ABCC6* gene in PXE was demonstrated in 2000 [2], [3], [5] and since then the clinico-genetic characteristics of the disease have been established [7], [8]. Interestingly, heterozygous carriers of *ABCC6* mutant alleles present an increased susceptibility to cardiovascular diseases [9], [10], [11]. The transcriptional regulation of the gene [6], [12], [13] and the biochemical characteristics of the ABCC6 protein have been well defined [14]. ABCC6 functions as an organic anion efflux pump [14], [15] transporting an as yet unidentified substrate(s) from the liver to the circulation. Because ABCC6 is predominantly found in liver and kidney and with little or no expression in tissues affected by PXE [8], [16], [17] this pathology appears to be systemic in nature. This implies the presence of an abnormal circulating molecule(s) that results from the failure of ABCC6 to export its substrate(s), ultimately promoting calcification in peripheral tissues. We've detected the presence of such circulating molecules in the serum of adult PXE patients through their effects on elastic fibers deposited in cultures [18] and others have reached similar conclusions using a mouse model [19], [20].

Since the first PXE-causing mutations were characterized [2], [3], [5], [21], the number of identified disease-causing variants has exceeded 300 [22]. Despite the large number of PXE-specific mutations that have been identified in *ABCC6*, no clear genotype-phenotype correlation has emerged [7], [21], [22]. However, a significant clustering of missense mutations was identified in domain-domain interfaces predicted from a homology model of the human ABCC6 protein [23]. Amino acid substitutions in large plasma membrane proteins such as ABCC6 generally result in decreased activity, major conformation changes, low level of plasma membrane targeting or a combination thereof. Therefore, studying the consequences of naturally occurring disease-causing missense mutations can provide important insights into the relationship between protein structure and function, which may later assist in the development of therapeutic applications. In recent years, several studies have shown that sodium 4-phenylbutyrate (4-PBA) can partially restore the cellular trafficking of a mutated ABCC7/CFTR (ΔF508) and parts of its cellular function were restored in both cultured cells and human cystic fibrosis patients [24], [25], [26], though how much phenotype improvement was achieved is less clear. Other proteins with disease-causing mutations were also subjected to 4-PBA treatment to improve their folding/trafficking like ABCA3 [27], LDL-receptor [28], the bile salt export pump/ABCB11 [29], [30], [31], ATP7B [32] and ATP 8B1 [33] and ATP-Sensitive Potassium Channel/ABCC8 [34]. 4-PBA is a butyrate analogue approved for clinical use in human with urea cycle disorders and thalassemia [35], [36], [37]. It is thought to interfere with the Hsc70 protein in endoplasmic reticulum (ER), allowing a proportion of misfolded proteins to escape association with the chaperon thus improving cellular trafficking [26], [38]. To capitalize on these precedent studies, we characterized the structural and functional consequences of selected PXE-causing missense mutations on ABCC6 transport activity, protein stability and conformation using *in vitro* assays. We also investigated for the first time, the *in vivo* stability and cellular location of WT and mutated ABCC6 in fully differentiated hepatocytes by transiently expressing the human mutant proteins in the liver of C57BL/6J mice. We focused on identifying those mutants with preserved transport activity and intracellular mistargeting. Such mutants are candidates for pharmacological rescue of their intracellular maturation. Indeed, we determined the potential for recovery of plasma membrane targeting of a transport-competent ABCC6-mutant in liver of C57BL/6J mouse after 4-PBA treatments.

## Materials and Methods

*The ABCC6 model*, previously generated by Fülöp *et al* [23] was analysed with the PyMOL Molecular Graphics System, Version 1.3, Schrödinger, LLC.

*Expression of ABCC6 variants in Sf9 insect cells, ATP binding, nucleotide trapping and vesicular transport* were performed as described in our previous papers [14], [39], [40], [41].

*Expression of ABCC6 variants in MDCKII cells* were achived by retroviral gene delivery as described [42].

### Liver-specific expression of ABCC6 variants in mice

The WT and mutant *ABCC6* cDNA constructs were sub-cloned into the pLIVE vector (Mirus Bio, Madison, WI) and expressed under the control of a liver-specific promoter. Plasmid DNA constructs were delivered by hydrodynamic tail vein injections [43], [44]. We used 3-month-old C57BL/6J wild type mice. The tail vein injections were performed with a 27-gauge needle with a volume of 1.5 to 2 ml of DNA in a solution of TransIT EE® following the manufacturers instructions (Mirus Bio Madison, WI). Mice were injected with 60 µg of a plasmid. At least 3 mice per mutant were injected.

### Animal

All mice were kept under routine laboratory conditions with 12 hours light-dark cycle with access *ad libitum* to water and standard chow. Mice were euthanized by standard CO_2_ procedures 24 hrs after tail vein injections. This study was approved by the Institutional Animal Care and Use Committee of the University of Hawaii.

### Immunoblotting and immunohistochemical staining of mouse liver samples

Multiple liver lobes were quickly harvested, placed in Optimum Cutting Temperature (OCT) compound and stored at −80°C. Immunofluorescent staining of liver samples was performed using 5 µm-thick frozen sections. The rat monoclonal anti-ABCC6 M6II-31 antibody (sc-59618) was used to specifically detect the human ABCC6. The rabbit polyclonal anti-Abcc6 antibody (S-20) was used to identify the mouse Abcc6. These primary antibodies were purchased from Santa Cruz Biotechnology Inc. (Santa Cruz, CA). The secondary antibodies were labeled with Alexafluor 488 and 568. We also used the rabbit polyclonal K-14 antibody, previously described [45]. A rabbit polyclonal antibody (Ab10286) from Abcam (Cambridge, MA) was used to detect the mouse Calnexin. The subcellular localization of the mouse Abcc6 and the human ABCC6 proteins was determined by imaging using an Axioscope 2 fluorescent microscope (Zeiss, Thornwood, NY). Fifty to one hundred individual images of cells for each mutant from at least 3 mice were collected and processed with Photoshop CS3 (Adobe, San Jose, CA) and then evaluated. Images were also analyzed using the ImageJ64 software (NIH) using the Graphic Dynamic Profiler tool.

### 4-PBA treatment of MDCKII cells and mouse

MDCKII cells were cultured as previously described [42] and in the presence of 1 mM 4-PBA (Tocris Biosciences, Ellisville, MO). Mice received 3 intraperitoneal injections of 4-PBA (100 mg/kg/day) prior to performing hydrodynamic tail vein injections.

## Results

**Figure 1:**
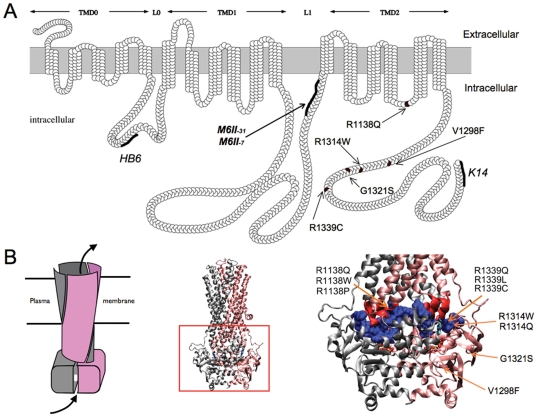
Membrane topology and homology model of the human ABCC6. **A:** The membrane topology was based on prediction previously described [53]. The locations of the various domains are indicated as horizontal arrows above the topology model. Various lines indicate the locations of the epitopes for the polyclonal antibody HB6 and K-14 and monoclonal antibodies M6II-7, M6II-31 utilized in the present study and arrows point to the position of the PXE missense mutations. **B:** The location of mutants in the three-dimensional homology model of the human ABCC6 protein [23] are shown. The entire model is shown with the two ABC halves of the molecule distinguished by grey and pink colors. The left panel is a schematic illustrating the domain swap characteristic of the ABC-protein superfamily. Protein segments of the ABC-domains forming the transmission interface are space-filled and colored in blue. Protein segments of the intracellular loops forming the transmission interface are colored in red.

The aim of our study was to characterize the structural and functional consequences of PXE-causing mutations in ABCC6 with a focus on the *in vivo* intracellular targeting of the human mutant proteins in mouse liver. Our main motivation was to identify disease-causing missense mutants with normal transport activity but with aberrant intracellular targeting. Such mutants were candidates for rescue of their intracellular maturation using chemical chaperons. We previously found an unequal distribution of ABCC6 missense mutations with high frequency on the ABC-ABC contact or in the transmission interface [23]. Therefore, we chose five missense mutations in these regions, V1298F and G1321S in the C-proxymal ABC domain and R1138Q, R1314W and R1339C in the transmission interface. The location of the mutated residues is illustrated on Figure 1, which depicts a model of the membrane topology of ABCC6 as well as a homology model of the protein. A N-terminal truncated mutant, del_1–277_ABCC6 (ΔABCC6) missing the TMD_0_ and L_0_ domains has also been constructed as a control (Figure 1).

### *In vitro* expression and functional characterization

**Figure 2:**
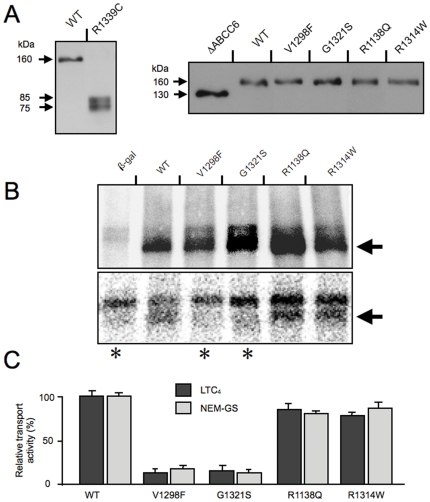
Biochemical characterization of the human WT and mutant ABCC6. **A:** Expression of ABCC6 variants in Sf9 insect cells as detected by western blotting. The WT ABCC6 and R1339C mutant were detected with the HB6 antibody, whereas the expression of ΔABCC6, V1298F, G1321S, R1138Q and R1314W was revealed with the M6II-7 antibody. **B:** Upper panel: specific MgATP-binding as revealed by photo-crosslinking of Sf9 membrane preparations with 8-N_3_-[a-^32^P] ATP under non-catalytic conditions (at 0°C).. b-gal: control membrane isolated from cells overexpressing b-galactoside. Lower panel: detection of the trapped ADP-vanadate complex (occluded nucleotide intermediate) as revealed by photo-crosslinking of the membrane preparation derived from Sf9 cells with 8-N_3_-[α-^32^P] ATP under catalytic conditions (at 37°C). b-gal: control membrane isolated from cells overexpressing b-galactoside. The arrow indicates the position of the labelled proteins **C:** Relative transport activities of ABCC6 and PXE-causing missense variants. Dark grey columns represent LTC4-transport activities whereas light grey columns indicate GS-NEM transport activities.

The wild type (WT) protein, ΔABCC6 and the 5 PXE-associated mutant ABCC6 proteins were expressed in Sf9 insect cells to first establish whether the mutants could be overexpressed. This was an important step to assess the overall stability of the ABCC6 mutants. We used western blotting with the M6II-7 monoclonal antibody and found that V1298F, G1321S, R1138Q, R1314W showed high levels of expression comparable to the WT ABCC6 at the expected molecular mass of ∼160 kDa (Figure 2A). The ΔABCC6 mutant was also stably overexpressed and displayed a molecular mass of ∼130 kDa, as expected. Surprisingly, the R1339C mutant could not be detected with the M6II-7 antibody. Using the polyclonal HB6 antibody, which recognizes a different region of ABCC6 (L0), we found that the R1339C variant was in fact degraded (Figure 2A). As both fragments (75 kDa and 85 kDa) reacted with the HB6 antibody recognizing an epitop in the N-proxymal part of the protein (for the location of the epitop, see Figure 1A), these were overlapping fragments indicating multiple degradation sites in the protein. As we were able to produce sufficient amounts of mutant ABCC6 proteins (except for R1339C), we analyzed the transporter characteristics of the mutants ABCC6. V1298F, G1321S, R1138Q, R1314W were capable of binding labeled MgATP (Figure 2A), indicating that the mutations did not alter significantly the conformation of the ATP-binding site(s). To determine if the PXE-mutants could form catalytic intermediate in which the gamma phosphate of ATP is cleaved, we studied the occluded nucleotide intermediate state (“vanadate trapping”) of the variants under catalytic conditions (37°C). Mutants V1298F and G1321S presented impaired activity while both R1138Q and R1314W mutants were capable of forming occluded nucleotide transitory complex as efficiently as the WT ABCC6 (Figure 2B). As we have previously demonstrated that glutathione-conjugated *N*-ethylmaleimide (NEM-GS) and Leukotriene C4 (LTC_4_) were both efficiently transported by ABCC6 *in vitro* (13), we continued the characterization of the mutants by measuring their transport activity with these two substrates. As shown on Figure 2C, mutants R1138Q and R1314W were able to actively transport both substrates at levels comparable to that of the WT ABCC6. In contrast, V1298F and G1321S exhibited little transport capacity as expected [14]. To complement these data, we also determined that the concentration-dependent LTC4 transport kinetics of R1138Q and R1314W was similar to the WT ABCC6 (not shown).

### Intracellular targeting of PXE-causing human ABCC6 in the liver of living mice

**Figure 3:**
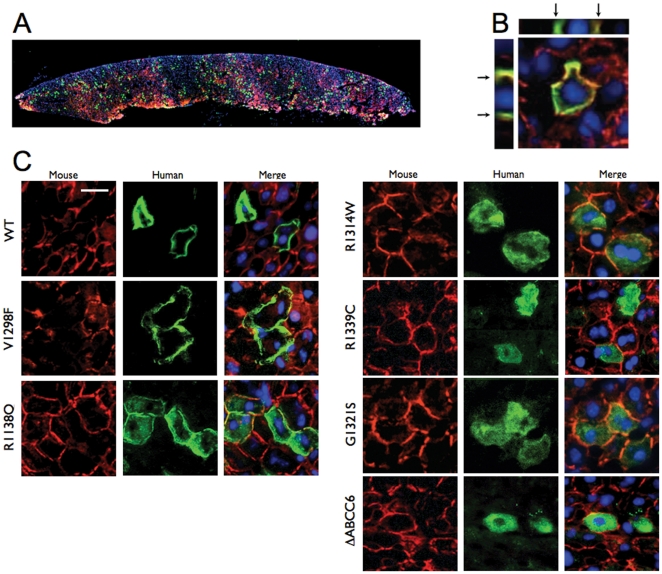
Intracellular localization of human ABCC6 variants expressed *in vivo* in mouse liver. The human and mouse ABCC6/Abcc6 were detected on frozen sections by immunofluorescence using the M6II-31 monoclonal antibody (green) and the S-20 polyclonal antibody (red), respectively, *Panel* **A:** A low magnification image shows the overall distribution of the human WT ABCC6 in a cross-section of single liver lobe. **B:** The basolateral plasma membrane localization of the WT ABCC6 expressed in mouse liver hepatocytes was confirmed by immunofluorescent imaging. The Z-stack cross-section images are also shown and arrows point to the basolateral membrane. **C:** Localization of the human WT and mutant ABCC6. Individual channels and the merged images of the endogenous Abcc6 (red) and the human ABCC6 variants (green) are shown. The scale bar represents 50 µm.

**Figure 4:**
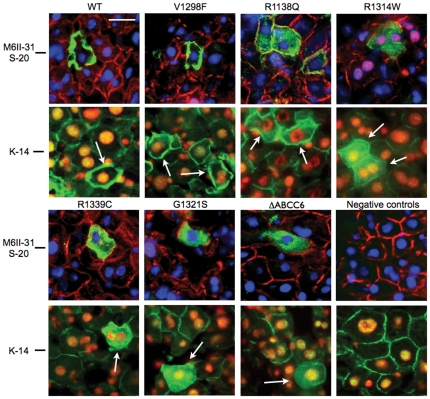
Intracellular localization of human ABCC6 variants expressed *in vivo* in mouse liver. The human ABCC6 variants were detected by immunofluorescence on liver frozen sections using the M6II-31 monoclonal antibody (upper panels) and the K-14 polyclonal antibody (lower panels). Subsequent frozen sections from the same blocks were used for the two different stainings. Note that K-14 polyclonal antibody recognizes both the human and mouse ABCC6/Abcc6. Immunostaining with K-14, which recognized the extreme C-terminal segment of ABCC6/Abcc6, indicated that full size proteins were expressed in mouse liver.

**Figure 5:**
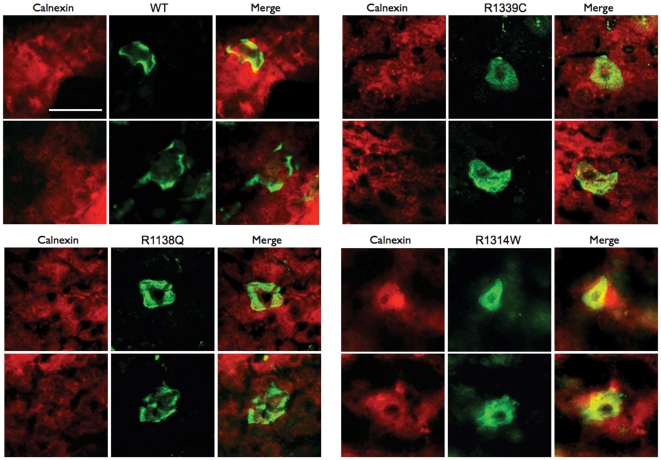
Co-localization of the endoplasmic reticulum (ER) marker Calnexin and selected ABCC6 mutants. The liver cells expressing the human ABCC6 variants (WT, R1339C, R1138Q and R1314W) were visualized by immunofluorescence on liver frozen sections using the M6II-31 monoclonal antibody (green) and a rabbit polyclonal antibody to the mouse ER marker Calnexin (red). Minimal co-localization (yellow) was seen between Calnexin and the WT and R1138Q mutant, while some spotty co-localization was visible for the R1339C mutant, primarily around the nuclei indicating partial ER retention or slower trafficking of this variant. The R1314W mutant showed the highest level of co-localization, which suggested that a large fraction of this protein is unable to leave the ER and translocate to the plasma membrane. Scale is shown on the upper left image (50 µm).

We studied the consequences of disease-causing mutations of ABCC6 in the liver of living mice, the organ where most of its physiological function is performed. We first sub-cloned the cDNAs of the 6 mutants and the WT protein into pLIVE vectors under the control of a liver-specific promoter consisting of the mouse albumin promoter and alpha fetoprotein enhancers. We then transiently expressed these cDNAs into the liver of adult normal mice using hydrodynamic tail vein injections (HTVI) [43]. Individual hepatocytes expressing the ABCC6 mutants were detected on frozen sections by immunofluorescence with the monoclonal antibody M6II-31 specific to the human ABCC6 along with a polyclonal antibody specific to the mouse Abcc6. Based on these immunostainings, we estimated that 5 to 10 percent of the mouse liver hepatocytes expressed detectable levels of the human ABCC6 (Figure 3A). The WT ABCC6 was fully integrated into the basolateral membrane of hepatocytes and co-localized with the mouse Abcc6 protein (Figure 3B). The ABCC6 mutants were also successfully expressed in mouse liver including R1339C despite the previously noted instability of this mutant in Sf9 cells. The cellular localization of all 6 mutants was determined by co-immunofluorescence staining (Figure 3C). The transport-incompetent mutant V1298F was the only mutant that showed a cellular localization identical to the human WT protein. In contrast, mutants R1314W, G1321S, R1314W, R1339C and ΔABCC6 were primarily located in the intracellular compartment with little or no plasma membrane localization (Figure 3C). The cellular targeting of R1138Q, however, showed an intermediary distribution both in the plasma membrane and in the intracellular space. To determine whether the immunofluorescent staining we obtained reflected the cellular localization of the integral protein for each ABCC6 variant, we performed immunostainings of the same liver samples with the polyclonal antibody K-14 that recognizes both the human ABCC6 and mouse Abcc6 proteins. This antibody was raised against the C-terminal end of the rat Abcc6 [45] We found identical intracellular staining patterns for each mutant (Figure 4), suggesting that the ABCC6 mutants were expressed as full size proteins in mouse liver. Next, we determined if mutants with residual transport activity and intracellular accumulation (R1138Q and R1314W) were retained in the endoplasmic reticulum. Mutant R1339C was also included into this experiment (our Sf9 exprression and transport assay was not suitable to determine its potential transport activity, see Figure 2A). We have addressed this question because it is established that the pharmacological rescue with 4-PBA can be successful in the case of ER-retained protein species [26], [38]. Figure 5 shows the results obtained of co-localization between Calnexin, a marker for the ER and variants R1138Q, R1314W and R1339C. The immunofluorescence images showed little of the WT protein resided in the ER. Interestingly, mutants R1318Q and R1339C also showed some co-localization with Calnexin while mutant R1314W was found to be predominantly associated with the ER.

Table 1 summarizes the structural and functional consequences of PXE-causing missense mutations in ABCC6 identified in this work.

**Table 1:** Function and intracellular localization of ABCC6 variants.

ABCC6 variant	Stability in Sf9	MgATP binding	ATPase catalytic intermediate	Transport activity(% of WT)	Plasmamembrane localization in mouse liver*	Intracellular localization in mouse liver*
WT	Stable	yes	yes	100%	+++++	−
ΔABCC6	Stable	n.d.	n.d.	<10%	−	+++++
R1138Q	Stable	yes	yes	∼85%	++	+++
V1298F	Stable	yes	no	<10%	+++++	−
G1321S	Stable	yes	no	<10%	−	+++++
R1314W	Stable	yes	yes	∼90%	+	++++(ER)
R1339C	Unstable	n.a.	n.a.	n.a.	−	+++++

n.d.: not determined.

n.a.: not applicable.

(ER): mostly retained with the endoplasmic reticulum.

*Plasma membrane and intracellular localization was determined independently by five of the co-authors using (+++++) for the plasma membrane and (−) for the intracellular localization of the WT and (−) for the plasma membrane and+++++for the intracellular localization of ΔABCC6, respectively.

### Pharmacological rescue of ABCC6 mutants

**Figure 6:**
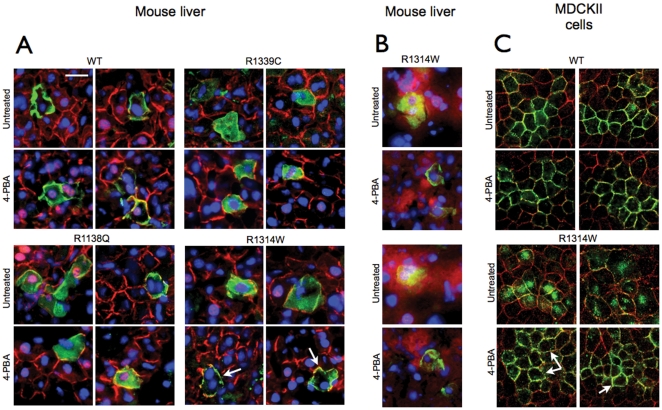
Pharmacological rescue of ABCC6 mutants. **A:** Mice were treated with 4-PBA prior to being subjected to HTVI with plasmids expressing the WT ABCC6 and mutants R1339C, R1138Q and R1314W. Immunostaining on frozen sections of mouse liver were performed to reveal both the mouse Abcc6 (red) and the human ABCC6 (green). The arrows show the improved plasma membrane localization of mutant R1314W as compared to the untreated controls. Each image was derived from a different mouse. The scale bar represents 50 µm. **B:** Co-localization between the endogenous ER marker Calnexin and mutant R1314W is indicated by yellow color. Treatment with 4-PBA facilitated the release of the latter mutant from the ER as indicated by the reduced co-localization. **C:** Effect of 1 mM 4-PBA treatment on the intracellular localization of R1314W in MDCKII cells as detected by immunofluorescence using the M6II-7 monoclonal antibody. The improved cellular localization of mutant R1314W is indicated by arrows.

As it was shown that 4-PBA can partially restore cellular trafficking (thus the function) of the ABCC7 cystic fibrosis mutant protein (ΔF508), we studied whether pre-treating mice with 4-PBA before HTVI could restore at least partial cellular trafficking of ABCC6 mutants that retained substantial transport activity. In the group of mutants we studied, R1138Q and R1314W showed near normal transport capacity despite incorrect cellular localization in the mouse liver. We also used the WT ABCC6 as a positive control as well as R1339C (though no information on its transport activity was obtained). Mice received 3 daily injections of 4-PBA before HTVI. We observed that the treatments did not alter the expression or plasma membrane localization of the WT ABCC6 and had no effect on the intracellular localization of R1138Q and R1339C. However, 4-PBA treated mice expressing R1314W showed a clearly improved membrane localization of this mutant as compared to untreated mice (Figure 6A and Supplementary Figure S1). This was confirmed by the lack of co-localization of R1314W with Calnexin (Figure 6B). For confirmation, we generated a MDCKII cell line overexpressing this mutant and found that R1314W was located in the intracellular space as was observed in the mouse liver. Treating these cells with 1 mM 4-PBA corrected the targeting of the mutant to the plasma membrane (Figure 6C).

These results not only showed that the structural consequence of the R1314W mutation could at least in part, be corrected by 4-PBA but also suggested that the incorrect intracellular trafficking of this mutant was likely due to ER retention and suggested protein misfolding.

## Discussion

We and others have accumulated a body of data that implicated ABCC6 as a new modulator of ectopic calcification in both human and mice [1], [2], [3], [4], [5]. This transmembrane protein is primarily expressed in liver, kidneys and intestine and transports unknown metabolite(s), which directly or indirectly control mineralization of dermal, ocular and cardiovascular tissues [46]. Mutations in the human *ABCC6* gene cause pseudoxanthoma elasticum, a recessive disorder [2], [3], [5], [22], [47]. The vast majority of *ABCC6* mutations are missense [7], [21], [22], [23] and several categories of missense substitutions can be distinguished based on the functional consequence of the amino acid replacement. Based on published data [26], [38], we have also anticipated that mutants with certain transport- and intracellular characteristics can be subjected to rescue by treatment with pharmacological compounds (called “chemical chaperons”). Therefore, the main objective of this study was to characterize the structural and functional consequences of *ABCC6* missense mutations *in vitro* as well as in an accurate *in vivo* model and to identify mutants suitable for rescue. ABC transporters are traditionally studied in cultures of MDCKII cells, but these are not ideal for studying hepatic proteins. Indeed, MDCKII cells are transformed cells derived from canine kidney tissues and do not reproduce the biology of the liver where ABCC6 is primarily expressed. A transgenic animal approach would be more accurate, yet it is time-consuming and poorly suited to the analysis of multiple protein variants. We have overcome these obstacles by demonstrating the feasibility of using HTVI to study normal and mutant forms of the human ABCC6 protein in the fully differentiated liver of a living mouse. This method delivers DNA to the liver very effectively [43], [44], [48] and ensures the selective hepatic expression of the WT human protein in mouse liver with an adequate basolateral targeting. We also analyzed an artificially truncated mutant, ΔABCC6 as similar truncated ABCC1 and ABCC2 proteins were found to be inactive and not integrated into the plasma membrane [41], [49]. The WT human protein co-localized with the mouse Abcc6 (Figure 3A, B) and thus validated our *in vivo* experimental model. Conversely, ΔABCC6 was completely absent from the plasma membrane arguing for the similar role of the TMD_0_ and L_0_ domains of ABCC6 with those of ABCC1 and ABCC2 in intracellular trafficking. Interestingly, two of the PXE-causing mutants we characterized, R1138Q and R1314W showed a near normal transport activity in *in vitro* assays, whereas mutants V1298F and G1321S were transport-deficient. We also observed that mutant R1339C could not be expressed in insect cells as an intact protein, but was successfully produced as full-length polypeptide in mouse liver following tail vein injection. The five mutants we examined demonstrated various degrees of intracellular accumulation *in vivo* ranging from WT behavior with normal plasma membrane position (V1298) to intracellular localization similar to ΔABCC6 (R1321S, R1339C or R1314W). R1138Q, however, showed an intermediate behavior with partial plasma membrane localization and a relatively abundant intracellular presence (Figure 3, see also Table 1). Because we have established that mutants G1321S, R1314W, R1339C and R1138Q were likely retained in intracellular compartments as non-degraded, full-size polypeptides (Figure 4), we used immunofluorescent imaging to identify the potential intracellular compartments involved. We have found that mutants R1138Q and R1339C showed some association with the ER suggesting partial ER retention. R1314W was mostly retained in the ER (Figure 5) indicating that the main consequence of pathologic amino acid substitutions in the human ABCC6 protein is the alteration of folding and/or trafficking. Though we have not specifically investigated it in this study, we found no evidence of protein accumulation in aggresomes or microtubule-organizing centers in images of hepatocytes expressing the ABCC6 mutants [50], [51], [52]. However, this would certainly be worth investigating, perhaps using MDCKII cells.

In summary, we have identified three possible consequences of PXE-causing mutations: 1) Transport deficiency due to the inability to use ATP (V1298F); 2) Altered folding and/or protein stability leading to intracellular retention (R1314W, R1339C, G1321S) in the ER or other cellular organelles, which could be pharmacologically corrected; and 3) Reduced trafficking efficiency with normal *in vitro* transport activity (R1138Q). We have summarized these results in Table 1. Despite the variability in the fate of these mutated proteins, all five mutations resulted in loss of physiological function, which to some extent, provide an explanation for the observed lack of phenotype–genotype correlation in PXE [21], [22]. In these experiments, we identified two mutant candidates to test whether a chemical chaperon could facilitate intracellular trafficking in mouse liver. Several studies have shown the possibility of rescue of membrane targeting of misfolded cystic fibrosis ABCC7/CFTR mutant (ΔF508) by treating either cultured cells or human patients with 4-PBA [24], [25], [26]. Similar results were obtained with disease-causing mutants ABCB11 in MDCKII cells [29]. Therefore, we explored the effect of 4-PBA in mice transiently expressing transport-competent ABCC6 mutants, R1138Q and R1314W as well as the R1339C variant and the WT protein for control purposes (Figure 6). The treatment had a remarkable effect on R1314W reverting the apparent ER retention of this mutant protein back to a near normal membrane localization in hepatocytes. A detailed analysis of liver sections from 4-PBA treated mice expressing R1314W is shown on Supplementary Figure S1. Treatment of MDCKII cells expressing the R1314W mutant with PBA resulted in a similar improvement (Figure 6C) thereby confirming the *in vivo* data.

In the present work we demonstrated the efficient association of *in vitro* studies with *in vivo* experiments to evaluate the consequences of missense ABCC6 mutations providing a unique insight into the intracellular processing of this human ABC transporter in physiological conditions similar to that of human primary hepatocytes. We also found that our integrated approaches constituted an appropriate base not only for testing pharmacological compounds with the ultimate aim of finding allele-specific therapeutic solutions for PXE but could also be a model for the systematic investigation of hepatic ABC transporters and other liver-specific membrane proteins.

## Supporting Information

Figure S1 **Effect of 4-PBA treatment on the intracellular distribution of R1314W ABCC6 mutant.** Mice were treated with 4-PBA prior to being subjected to HTVI with plasmids expressing ABCC6 R1314W. Immunostaining were performed on liver frozen sections from mice exposed to 4-PBA (rows 3 and 4) or from untreated animals (rows 1 and 2). Each row represents a different animal. Histograms were generated by ImageJ64 software using the Graphic Dynamic Profiler tool. Arrows indicate the lines of signal sampling.
(TIF)
