# Supplementary figures and images for: Expression and In Vivo Rescue of Human ABCC6 Disease-Causing Mutants in Mouse Liver
Source: PLoS One. 2011 Sep 14;6(9):e24738. doi: 10.1371/journal.pone.0024738 (PMC3173462; doi:10.1371/journal.pone.0024738)

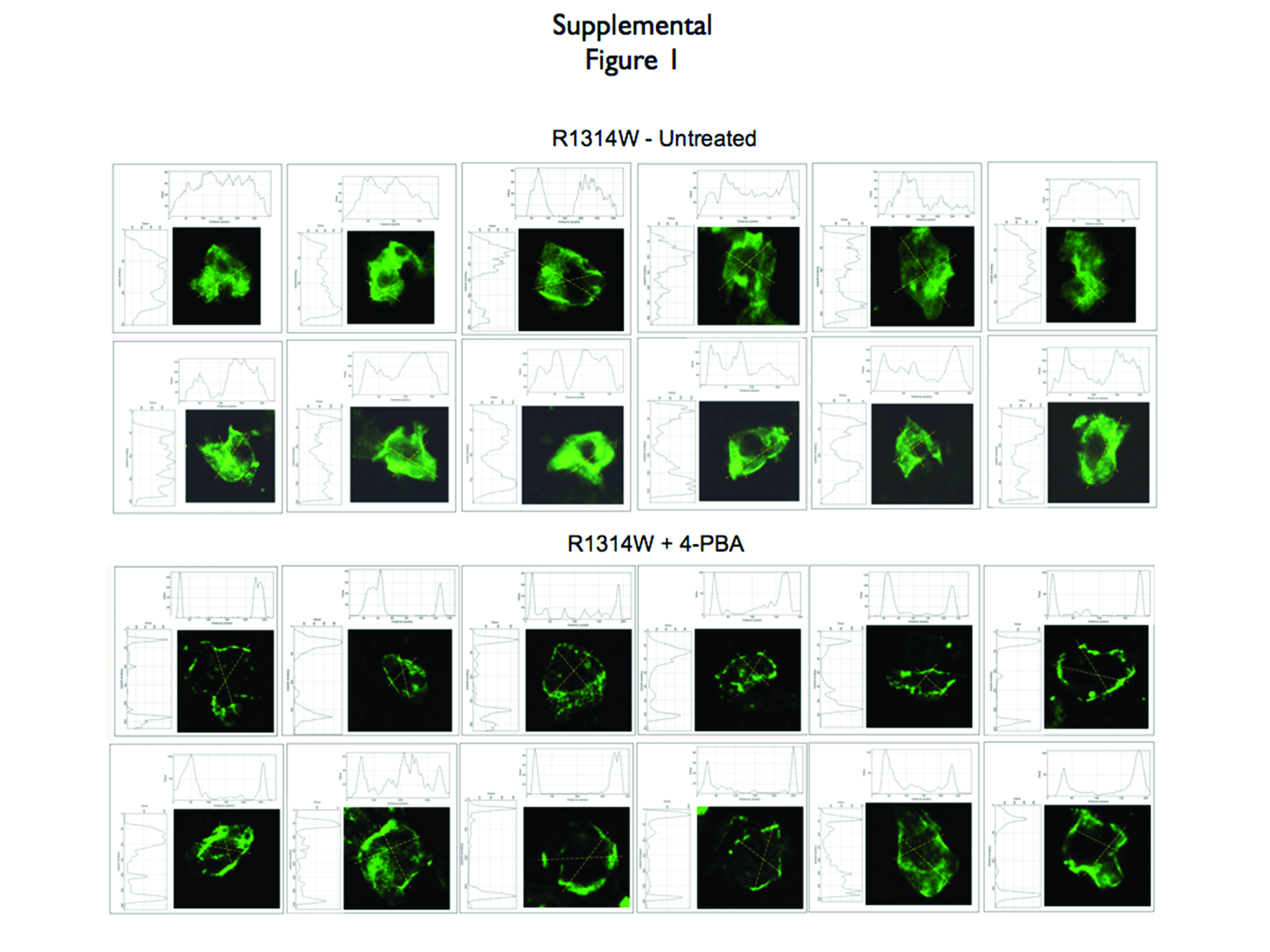

Supplement: Figure S1 — Effect of 4-PBA treatment on the intracellular distribution of R1314W ABCC6 mutant. Mice were treated with 4-PBA prior to being subjected to HTVI with plasmids expressing ABCC6 R1314W. Immunostaining were performed on liver frozen sections from mice exposed to 4-PBA (rows 3 and 4) or from untreated animals (rows 1 and 2). Each row represents a different animal. Histograms were generated by ImageJ64 software using the Graphic Dynamic Profiler tool. Arrows indicate the lines of signal sampling. (TIF) [file pone.0024738.s001.tif]
